# Does a Hypertrophying Muscle Fibre Reprogramme its Metabolism Similar to a Cancer Cell?

**DOI:** 10.1007/s40279-022-01676-1

**Published:** 2022-04-23

**Authors:** Henning Wackerhage, Ivan J. Vechetti, Philipp Baumert, Sebastian Gehlert, Lore Becker, Richard T. Jaspers, Martin Hrabě de Angelis

**Affiliations:** 1 Exercise Biology Group, Department of Health and Sports Sciences, Technical University of Munich, Munich, Germany; 2 Department of Nutrition and Health Sciences, College of Education and Human Sciences, University of Nebraska-Lincoln, Lincoln, NE USA; 3 Department of Biosciences of Sports, Institute for Sports Science, University of Hildesheim, Hildesheim, Germany; 4 Institute of Experimental Genetics, Helmholtz Zentrum München, German Research Center for Environmental Health, Munich, Germany; 5 Laboratory for Myology, Behavioural and Movement Sciences, Amsterdam Movement Sciences, Vrije Universiteit Amsterdam, Amsterdam, The Netherlands; 6 German Center for Diabetes Research (DZD), Neuherberg, Germany; 7 Chair of Experimental Genetics, TUM School of Life Sciences, Technische Universität München, Freising, Germany

## Abstract

In 1924, Otto Warburg asked “How does the metabolism of a growing tissue differ from that of a non-growing tissue?” Currently, we know that proliferating healthy and cancer cells reprogramme their metabolism. This typically includes increased glucose uptake, glycolytic flux and lactate synthesis. A key function of this reprogramming is to channel glycolytic intermediates and other metabolites into anabolic reactions such as nucleotide-RNA/DNA synthesis, amino acid-protein synthesis and the synthesis of, for example, acetyl and methyl groups for epigenetic modification. In this review, we discuss evidence that a hypertrophying muscle similarly takes up more glucose and reprogrammes its metabolism to channel energy metabolites into anabolic pathways. We specifically discuss the functions of the cancer-associated enzymes phosphoglycerate dehydrogenase and pyruvate kinase muscle 2 in skeletal muscle. In addition, we ask whether increased glucose uptake by a hypertrophying muscle explains why muscularity is often negatively associated with type 2 diabetes mellitus and obesity.

## Key Points

**Table:** 

Nearly 100 years ago, Otto Warburg discovered that cancer cells reprogramme their metabolism by increasing glucose uptake and lactate synthesis in the presence of oxygen, which is termed the Warburg effect.
Currently, we know that the metabolic reprogramming in proliferating healthy and cancer cells helps to generate glycolytic intermediates and other metabolites as substrates for anabolic reactions to build biomass.
Myc, hypoxia-induced factors and Pi3k–Akt–mTor not only regulate metabolic reprogramming in cancer but are also signalling molecules that are activated by resistance training and stimulate muscle hypertrophy.
For every gram of biomass that a muscle builds, it needs to take up 1 g of small-molecule substrates such as glucose, amino acids and other molecules. This uptake of glucose and other metabolites may explain why muscular organisms or resistance-trained individuals are often lean and have good insulin sensitivity.

## Introduction

In 1924, Otto Warburg published a paper on the metabolism of cancer cells in *Naturwissenschaften*. In it, he asked “How does the metabolism of a growing tissue differ from that of a non-growing tissue?” [1]. In this paper, Warburg demonstrated in vitro [1] and 3 years later in vivo [2] that cancer cells reprogramme their metabolism. Key features of this metabolic reprogramming are that cancer cells take up more glucose and synthesise more lactate in the presence of oxygen than non-growing cells and organs. Ephraim Racker later termed such aerobic glycolysis the “Warburg effect” to contrast it with the “Pasteur effect,” which is anaerobic glycolysis [3].

In this review, we first discuss the Warburg effect in cancer and how it helps to provide substrates for anabolism and for the generation of biomass. We then review evidence that hypertrophying muscles reprogramme their metabolism similarly to cancer cells. Third, we discuss the potential benefits of such metabolic reprogramming for the prevention and treatment of type 2 diabetes mellitus and obesity.

## How Do Healthy and Cancerous Proliferating Cells Reprogramme Their Metabolism?

Most but not all healthy and cancerous proliferating cells take up more glucose as well as synthesise more lactate in the presence of oxygen than non-proliferating cells [4, 5]. Otto Warburg hypothesised that this was because of a mitochondrial defect causing cancer cells to rely on glycolysis for ATP resynthesis [6]. However, many cancers have functioning mitochondria [7] and thus a defective oxidative phosphorylation does not generally explain increased glycolytic flux in cancer.

**Fig. 1:**
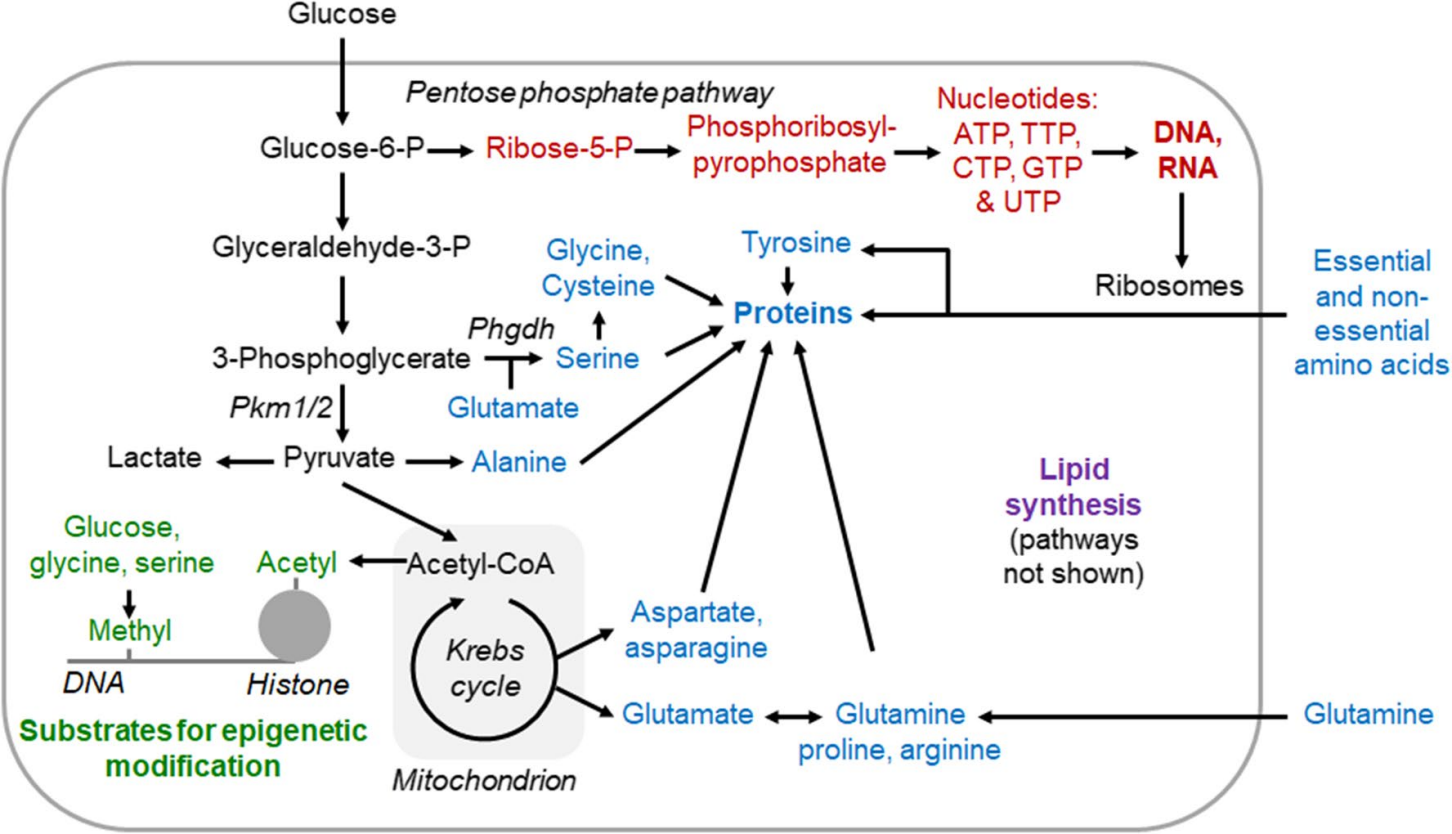
Schematic showing some connections in-between energy metabolism and anabolism modified after [5]. In proliferating cells, glycolysis is typically upregulated, and glycolytic intermediates and other energy metabolites flow more into anabolic reactions such as nucleotide-DNA/RNA synthesis (red), amino acid-protein synthesis (blue), lipid synthesis (purple) and the synthesis of small groups used for epigenetic modification (green). Note that not only glucose but also glutamine is taken up at a high rate by rapidly proliferating cells [8, 10]. For further information, for example, on substrate synthesis for epigenetic modification, see DeBerardinis and Chandel [5]. *CTP* cytosine triphosphate, *GTP* guanosine triphosphate, *P* phosphate, *Phgdh* phosphoglycerate dehydrogenase, *Pkm1/2* pyruvate kinase muscle isoforms 1 and 2, *TTP* thymine triphosphate, *UTP* uracil triphosphate

So, what is the Warburg effect good for? Otto Warburg and subsequent scientists until the late 1990s were unable to answer this question satisfactory. Research since then has shown that a key function of the metabolic reprogramming in cancer is to generate glycolytic intermediates and other energy metabolites as substrates for anabolism, which is the part of metabolism that synthesises “cellular components from precursors of low molecular weight” (International Union of Pure and Applied Chemistry definition 1997). More specifically, proliferating cells take up more glucose, glutamine [8] and other small molecules, and then channel these molecules into glycolysis or other energy metabolic reactions, which are “feeder pathways” for anabolic reactions. These anabolic reactions include the synthesis of nucleotides for DNA and RNA, the synthesis of non-essential amino acids for protein synthesis and the synthesis of other macromolecules. How energy metabolism is connected to anabolism is for example illustrated by the IUBMB-Nicholson Metabolic Pathways Chart [9]. An overview over some key reactions involved in the metabolic reprogramming of proliferating and/or growing cells is illustrated in Fig. 1.

More recent research shows that the metabolic reprogramming varies from cancer to cancer, suggesting that it is not a rigid programme. For example, 149 out of 240 cancers overexpressed glycolytic genes [11]. In a more comprehensive analysis from 2016, the researchers compared the expression of metabolic genes across 20 types of cancer with their expression in the corresponding healthy tissue. They found that 14 cancers (70%) overexpressed purine synthesis genes, ten (50%) overexpressed DNA synthesis genes, seven (35%) overexpressed oxidative phosphorylation genes, and only five (25%) overexpressed glycolysis and gluconeogenesis genes [12]. The limitation of these metabolic gene expression analyses is that they depend on how the criteria for “overexpression” are set and that they do not measure actual changes in metabolic flux in-between proliferating and non-proliferating cells. Nonetheless, they suggest that the metabolic reprogramming of proliferating cells varies. 

In summary, a key function of the metabolic reprogramming of proliferating cells is to provide substrates for anabolic pathways that generate the biomass necessary for cell proliferation. Otto Warburg already in 1924 stated that such reprogramming was not unique to cancer but also occurred for example in the growing chick embryo [13]. Several reviews discuss specific aspects of metabolic reprogramming in cancer such as the regulation of the metabolic reprogramming, the metabolic pathways involved (Fig. 1), the function of metabolic reprogramming beyond energy metabolism and anabolism, research methods to analyse metabolic reprogramming as well as unanswered questions [4, 5, 14].

## Is There Evidence That Metabolic Reprogramming Occurs When Muscle Fibre Hypertrophy?

Muscle fibres are, in contrast to proliferating cells, non-proliferating syncytia (i.e. multi-nuclear cells) with thousands of nuclei and muscle fibres can reach a length of up to ≈ 20 cm in humans [15]. However, given that proliferating cells reprogramme their metabolism to channel more energy metabolites into anabolism, and given that muscle hypertrophy relies on anabolism, an intriguing question is: Do hypertrophying, post-mitotic muscle fibres reprogramme their metabolism similar to proliferating cells?

Before answering this question, we highlight a key difference in terms of anabolism between proliferating cells and hypertrophying muscle fibres. Proliferating cells need to replicate their entire genome during each cell cycle. This means that each proliferating human cell will synthesise 6.4 billion nucleotides plus nucleotides for mRNA and ribosomal biogenesis before dividing into two daughter cells. In contrast, a hypertrophying muscle fibre “only” synthesises nucleotides for mRNA and ribosome biogenesis [16] as it has “outsourced” replication and the generation of new myonuclei to proliferating satellite cells. The fact that ribose, the pentose sugar in RNA, and deoxyribose, the pentose sugar in DNA, are primarily synthesised from glucose [17] is one reason for why proliferating cells take up more glucose. However, the ribosome biogenesis of hypertrophying muscle fibres [16] will also require glucose to synthesise the nucleotides and rRNA from which ribosomes are made. Hosios et al. have directly compared the contribution of radioactive or stable isotope tracers such as ^14^C/^13^C-glucose or ^14^C/^13^C-glutamine to the cell mass of proliferating C2C12 myoblasts and differentiated C2C12 myotubes [10]. They found that glucose and glutamine contributed 15% and 8% to cell mass in proliferating C2C12 myoblasts in a steady state and 6% and 3% to cell mass in differentiated but not hypertrophy-stimulated C2C12 myotubes after 6 days of incubation, respectively [10]. In summary, proliferating and hypertrophying cells both generate biomass via anabolic reactions but only proliferating cells synthesise DNA for replication.

Back to the question whether hypertrophying post-mitotic muscle fibres reprogramme their metabolism similar to proliferating cells. First, indirect evidence for metabolic reprogramming during muscle hypertrophy comes from studies that show that cancer metabolic reprogramming factors such as the transcription factor *Myc* [18] and hypoxia-induced factors, which typically regulate glycolytic genes [19], become more expressed in overloaded hypertrophying mouse muscle (*Myc* and *Hif1a* [20]) or in human muscle after a bout of resistance exercise (*MYC* and *EPAS1* [21]). Moreover, Pi3k–Akt–mTor signalling, which also regulates metabolic reprogramming in cancer [5], is activated as judged by the phosphorylation of mTORC1 proteins such as S6K1 [22–25]. However, whilst for example Myc drives the expression of lactate dehydrogenase A (gene *Ldha*) in cancer, only *MYC* expression but not *LDHA* expression increases in human muscle after resistance exercise [21]. Although *MYC* is probably not a driver of *LDHA* expression in human muscle, adeno-associated mediated Myc overexpression in the mouse gastrocnemius is sufficient for ribosomal biogenesis and increased protein synthesis [26]. As ribosomes comprise ≈ 60% rRNA, this requires first synthesising nucleotides from a purine or pyrimidine, sugar and phosphate followed by rRNA biosynthesis and ribosome biogenesis. This requires the regulation of metabolic pathways such as the pentose phosphate pathway. In summary, regulatory molecules such as Myc, Hif proteins and Pi3k–Akt–mTor signalling become more expressed or activated after resistance training. Especially for Myc and Pi3k–Akt–mTor signalling (see below), there is evidence that this also reprogrammes the metabolism of hypertrophying muscle fibres.

Second, inhibiting glycolysis with 500 mg/kg of 2-deoxyglucose reduces basal protein synthesis of untreated and electrically stimulated rat gastrocnemius muscle 6 h after stimulation when compared with untreated controls [27]. This suggests that glycolytic flux limits protein synthesis, which is the primary cause of muscle hypertrophy.

Further evidence for the idea that hypertrophying myotubes reprogramme their metabolism comes from a study where the researchers compared cultured control myotubes to insulin-like growth factor (IGF-1)-transfected myotubes. The IGF-1-transfected C2C12 myotubes had a 2.7-fold higher rate of protein synthesis and were > 50% wider than control myotubes, indicating that IGF-1 had stimulated myotube hypertrophy. The activity of lactate dehydrogenase (gene *Ldh*) in the IGF-1-transfected myotubes was 3.2 times higher and the concentration of lactate was 2.4 times higher than in the control myotubes [28], suggesting that a Warburg-like metabolic reprogramming was triggered by IGF-1. Moreover, IGF-1 activates the Pi3k–Akt–mTor pathway, which helps to reprogramme metabolism in cancer [5]. Muscle hypertrophy achieved by synergist ablation also increases glycolytic flux by ≈ 60% in the absence of insulin in overloaded incubated soleus muscles when compared with control solei [29]. In a more recent publication, the authors confirmed that an overload roughly doubled plantaris glucose uptake, increased lactate secretion by ≈ 50% (but no effect on glycolytic flux) and activated the pentose phosphate pathway as evidenced for example by increased glucose-6 phosphate dehydrogenase protein levels [30]. Moreover, we recently demonstrated that the expression of *G6pd*, which encodes glucose-6 phosphate dehydrogenase, the rate-limiting gene of the pentose phosphate pathway, is upregulated together with other genes of the pentose phosphate pathway in mechanically overloaded mouse plantaris muscles [31]. The pentose phosphate pathway is active in cancer [32] and synthesises nucleotides for DNA, RNA and ribosome biogenesis (ribosomes primarily consist of ribosomal RNA).

Akt1 is a serine/threonine kinase and member of the Pi3k–Akt–mTor pathway. In vivo, inducing the expression of constitutive active *Akt1* in mouse muscle for 2 weeks nearly doubled mean type 2B muscle fibre size from 1406 ± 21 µm^2^ to 2788 ± 139 µm^2^ [33]. The muscles of these mice also expressed more hexokinase (gene *Hk2*), phosphofructokinase (gene *Pfk*), lactate dehydrogenase (gene *Ldha*) and the cancer-specific isoform 2 of pyruvate kinase (gene *Pkm2*) [33, 34].

Additional supporting evidence that Pi3k–Akt–mTor signalling not only promotes muscle hypertrophy but also metabolic reprogramming comes from mice where the mTORC1 inhibitor Nitrogen Permease Regulator 2-Like Protein (*Nprl2)* is knocked out. Again, this causes muscle fibre hypertrophy and induces aerobic glycolysis as judged by a three-fold higher expression of *Slc2a1,* which encodes the glucose transporter *Glut1*, and two-fold higher expressions of *Hk2,* encoding hexokinase 2, and *LdhB,* encoding lactate dehydrogenase B. Moreover, many amino acid metabolism-regulating genes change their expression in muscles of *Nprl2* knock-out mice [35].

Finally, inhibiting mTORC1 with rapamycin (termed everolimus in this paper [36]) in C2C12 myotubes changed the concentrations of many glycolytic intermediates and of metabolites of the pentose phosphate pathway. Moreover, rapamycin reduced the expression of glucose handling and glycolytic genes such as *Slc2a1* and *Hk1,* which encode hexokinase 1, and *Pfkm1,* which encodes the rate-limiting enzyme of glycolysis, phosphofructokinase. Collectively, the metabolomics, gene expression and enzyme activity analyses suggest that mTORC1 blockage with rapamycin causes the opposite of a cancer-like metabolic reprogramming [36].

However, metabolic reprogramming during muscle hypertrophy is not only induced by IGF-1–Pi3k–Akt–mTor signalling. For example, *Mstn* knockout mice have more type 2B fibres and an increased glycolytic capacity [37]. It is unclear how these hypertrophy regulators modulate the fibre-type distribution and whether the increased expression of glycolytic enzymes is due to the induction of a Warburg effect-related gene expression or simply due to a slow-to-fast muscle fibre type shift. Interestingly, more glycolytic type 2 muscle fibres also hypertrophy to a greater extent than less glycolytic type 1 muscle fibres after resistance exercise [38]. Moreover, the inverse relationship between oxidative metabolism and muscle fibre size as shown between muscle fibres within a muscle as well as between fibres of different species [39] indicates a tight association between muscle size and glycolytic and oxidative metabolism. Paradoxically, slow high oxidative muscles possess a higher content of components of the protein synthesis machinery (i.e. a higher content of myonuclear density and ribosomes) than fast glycolytic muscle fibres [39, 40]. Despite the lower potential for protein synthesis, the hypertrophic potential of fast glycolytic muscle fibres is higher and currently there is no satisfactory explanation for this [39]. One hypothesis could be that the higher glycolytic capacity of type 2 fibres increases the capacity for a Warburg-like metabolic reprogramming, and thereby facilitates muscle hypertrophy.

Another study investigated the effect of testosterone, a male sex hormone that increases muscle fibre size [41], on rat myotubes. Testosterone induced hypertrophy and increased the activity of the glycolytic enzymes hexokinase and phosphofructokinase in a dose-dependent manner [42], again consistent with a Warburg-like metabolic reprogramming.

In our own research, we have studied two enzymes associated with metabolic reprogramming in cancer. The first is pyruvate kinase muscle (gene *PKM*; EC 2.7.1.40), which catalyses the last step of glycolysis from phosphoenolpyruvate to pyruvate. PKM is alternatively spliced into Pkm1 and Pkm2 variants. Pkm2 is especially expressed in cancer and proliferating cells and has non-glycolytic regulatory functions [43]. Alternative Pkm splicing also occurs in skeletal muscle. For example, embryonal muscle expresses Pkm2, which is associated with proliferation [44] and then shifts to Pkm1 in adult muscle [45]. In vitro, the knock down of Pkm2 reduces myotube size whereas the knock down of Pkm1 increases C2C12 myotube size, suggesting that a high Pkm2/Pkm1 ratio promotes myotube hypertrophy [46]. However, we know little about how Pkm splicing and activity are regulated in a hypertrophying muscle. A phosphoproteomics study has revealed that Pkm is phosphorylated at multiple sites in skeletal muscle [23] and we found that the PKM2 isoform is more expressed in fast glycolytic muscle fibres and increases after weeks of resistance training [46]. Moreover, reducing Pkm2 by shRNA or the Pkm2 inhibitor shikonin in C2C12 myoblasts reduced C2C12 myoblast proliferation, but knocking out *Pkm2* in Pax7-positive satellite cells did not impair regeneration after a muscle injury [44]. Together this suggests that the Pkm2 isoform can help to promote anabolism.

A second enzyme that is associated with metabolic reprogramming in cancer is Phgdh (EC 1.1.1.95). It diverts the glycolytic metabolite 3-phospho-d-glycerate from energy metabolism into the serine biosynthesis pathway. An unbiased RNA interference screen of metabolic genes has identified Phgdh as an enzyme that limits proliferation of breast cancer cells [47]. In muscle, *Phgdh* expression increases in mouse muscle that hypertrophies after synergist ablation [20, 31]. *Phgdh* expression also transiently increases in response to β_2_-agonist stimulation [48] but not after human resistance exercise [21]. In vitro, Phgdh knock down reduces C2C12 myotube size, suggesting that the increased Phgdh activity promotes hypertrophy [49].

Finally, Japanese researchers have characterised the metabolome, gene expression and proteome response of C2C12 myotubes to 2-Hz and 20-Hz electrical pulse stimulations. In this model, 20-Hz stimulation is presumably a model for resistance exercise-induced muscle hypertrophy. The combined data suggest that 20-Hz stimulation activated the pentose phosphate pathway, which helps to synthesise nucleotides for RNA and DNA synthesis. Again, this suggests a metabolic reprogramming that goes beyond energy metabolism [50].

Taken together, there is scientific “smoke” that a Warburg effect-like metabolic reprogramming occurs at least in some models of skeletal muscle hypertrophy. Whilst there is no general upregulation of glycolytic and cancer metabolism-associated genes in a resistance-trained human muscle [21], in some situations, glycolytic enzyme expression increases and some cancer reprogramming-associated genes such as *Pkm2* and *Phgdh* can limit muscle hypertrophy. Furthermore, each kilogram of fat-free, human muscle dry mass comprises ≈ 715 g of protein, ≈ 4 g of RNA, ≈ 2 g of DNA [51] and consequently ≈ 279 g of other molecules such as phospholipids in membranes. Thus, whilst nucleotide synthesis must occur for ribosome biogenesis [16] and whilst proliferating satellite cells will synthesise nucleotides for replication, the quantitatively most important biomass-generating process will be protein synthesis from amino acids that are taken up or that are synthesised by the muscle fibre from precursors.

Researchers should now use the full tool kit of modern metabolic research to quantitatively characterise the metabolic reprogramming that occurs during muscle hypertrophy. Importantly, researchers should verify that the metabolic reprogramming during muscle hypertrophy is not just an adaptation of energy metabolism but that it serves functions such as synthesising substrates for anabolic reactions as in cancer [5]. Finally, we have not discussed the metabolic reprogramming of proliferating satellite cells [52] in a hypertrophying muscle as satellite cells only contribute a small fraction of the volume of a muscle.

## Does Metabolic Reprogramming During Muscle Hypertrophy Affect Our Health?

A high glucose uptake by tumour cells was one of the key original observations of Otto Warburg [1, 2, 53]. In relation to muscle hypertrophy, the question arises: does a hypertrophying muscle fibre similarly take up more glucose and does it channel some of that glucose into anabolic reactions? If that was the case, then muscle hypertrophy should have positive health effects because a higher glucose uptake by hypertrophying muscles would improve glycaemic control and reduce the amount of glucose available for lipid synthesis by adipose tissue. If these reactions removed a sufficiently high amount of glucose and other small molecules from the circulation, then muscle hypertrophy could help to prevent or treat diabetes and obesity.

**Fig. 2:**
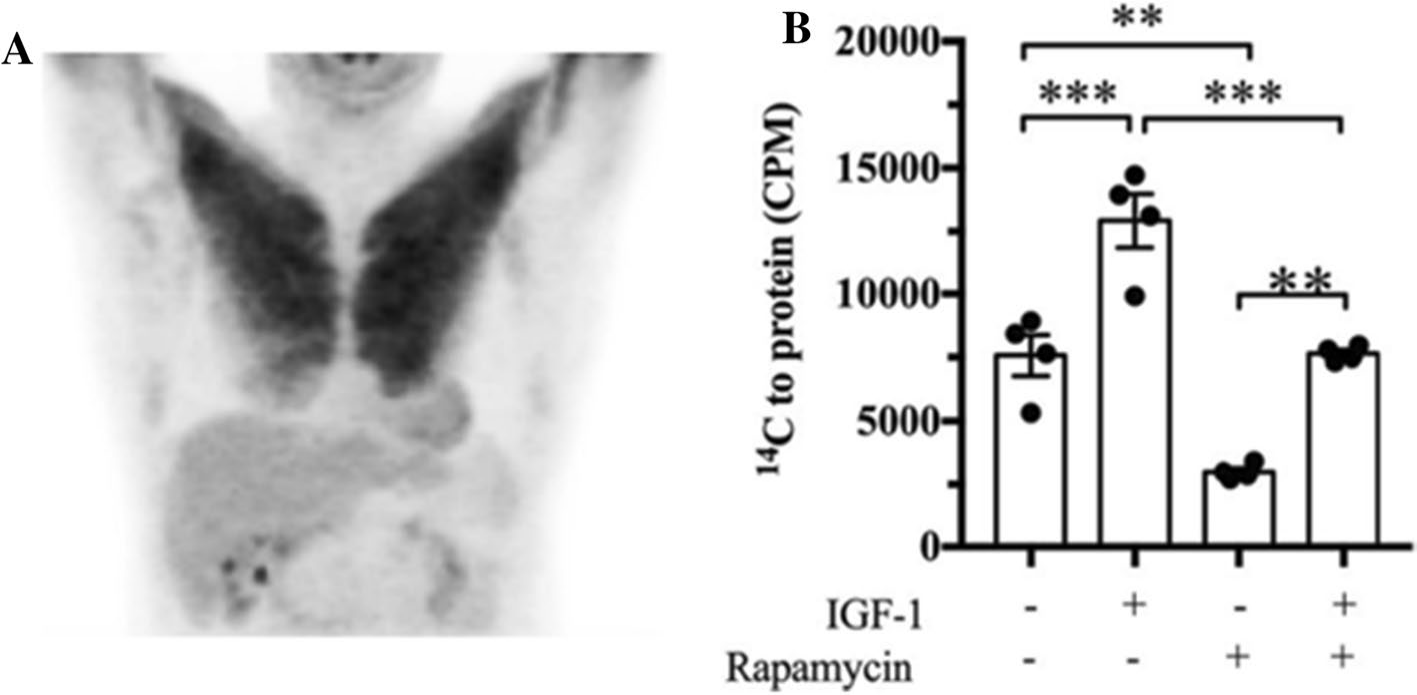
**A** Fluorodeoxyglucose uptake of a pectoralis muscle 24 h after strenuous upper limb exercise [54]. **B** Incorporation of glucose-derived ^14^C into muscle protein. In this experiment, we incubated C2C12 myotubes with radioactive ^14^C-glucose and treated them with combinations of 100 ng mL^−1^ of insulin-like growth factor (IGF-1) or 100 ng mL^−1^ of rapamycin. We observed that ^14^C from glucose ended up in protein and that IGF-1 increased the incorporation of ^14^C into protein significantly by ≈ 71% [49]. Note that the data in B are from a not yet peer-reviewed preprint [49]. *CPM* counts per minute

There is indeed evidence that hypertrophying muscles take up more glucose and that this improves glycaemic control and reduces white adipose tissue. Figure 2A shows the 18F-fluoro-2-deoxy-d-glucose positron emission tomography scan of a patient who had performed “strenuous upper limbs exercise [presumably resistance exercise] 24 h prior to the imaging”. The scan suggests that the pectoralis muscle of the patient takes up a high amount of glucose 1-day post-exercise [54]. The caveat, however, is that we are unable to say whether the taken-up glucose is channelled into anabolism or is simply used to resynthesise the glycogen that was used during exercise. In another study, a Copenhagen team asked healthy and type-2 diabetic volunteers to perform a 6-week, one-sided leg resistance training. After the training, they performed an isoglycaemic-hyperinsulinemic clamp procedure and found that the resistance-trained leg took up ≈ 25% (healthy) and ≈ 10% more glucose (type 2 diabetes, both *p* > 0.05) than the untrained leg [55]. Moreover, in mice, synergist ablation-induced soleus hypertrophy increased both glucose uptake and glycolytic flux in lean (especially at insulin concentrations < 5 nmol/L) and obese mice at all insulin concentrations when compared with the untreated control soleus [29]. Additionally, overloaded, hypertrophying mouse plantaris muscles take up ≈ 60% more glucose than control plantaris. In *Slc2a4* (encoding the glucose transporter Glut4) knock-out mice, the difference is even greater, as the glucose uptake of the hypertrophying plantaris is similar to the wild-type hypertrophying plantaris but glucose uptake into the non-hypertrophying control plantaris is decreased [56]. Collectively, these studies suggest that resistance-trained and/or hypertrophying mouse and human muscles take up more glucose than untrained or non-hypertrophying muscles. But why? Is it just to replenish glycogen or is a fraction of the glucose channelled into anabolism?

To specifically test whether muscle can channel glucose into anabolism and specifically amino acid and protein synthesis, we incubated C2C12 myotubes with radioactive ^14^C-glucose, extracted protein and used a scintillation counter to investigate whether ^14^C in glucose can be incorporated into protein. This experiment confirmed that glucose-derived ^14^C can be incorporated into muscle protein and that hypertrophy stimulation by IGF-1 increases the rate of ^14^C incorporation into protein presumably via an ^14^C-glucose → ^14^C-glycolytic intermediate → ^14^C-non-essential amino acid → ^14^C-protein pathway. Conversely, inhibiting mTORC1 with rapamycin reduced ^14^C incorporation into protein ([49] Fig. 2B). The fact that C2C12 myotubes can incorporate glucose-derived carbon into cell mass was also observed in another study [10]. In summary, hypertrophying muscles take up glucose for at least 1 day after a bout of resistance exercise and the stimulation of hypertrophy by IGF-1 increases the incorporation of ^14^C from glucose into myotube protein, which is consistent with the idea that a hypertrophying muscle channels more glucose and energy metabolites into anabolism.

Does a Warburg-like metabolic reprogramming of hypertrophying muscles improve insulin resistance? If resistance exercise stimulated glucose uptake to channel glucose-derived energy metabolites into anabolism, then resistance exercise should improve insulin resistance. Indeed, meta-analyses conclude that resistance training improves glycaemic control in individuals at risk for diabetes [57] and in patients with type 2 diabetes [58]. However, it is unclear whether the glycaemia-improving effects are explained fully by increased glycogen resynthesis or whether some of that glucose is channelled into anabolism. Several studies suggest that muscle hypertrophy not triggered by glycogen-reducing exercise can increase glucose uptake and that this is anti-diabetic. First, testosterone stimulates GLUT4 expression and GLUT4 membrane localisation in cultured primary rat myotubes [42]. This should increase glucose uptake, too, but the authors did not measure this. Second, myostatin receptor inhibition not only increased muscle mass but also prevents diabetes in *A-ZIP/F1* mice that normally develop diabetes [59]. Third, inducing muscle hypertrophy by expressing constitutively active Akt1 in muscle prevented elevated blood glucose and insulin concentrations in mice on a high-fat and high-sugar diet, again demonstrating the anti-diabetic effects of muscle hypertrophy [33]. In summary, muscle hypertrophy improves glycaemia even if it is not triggered by glycogen-reducing resistance exercise.

**Fig. 3:**
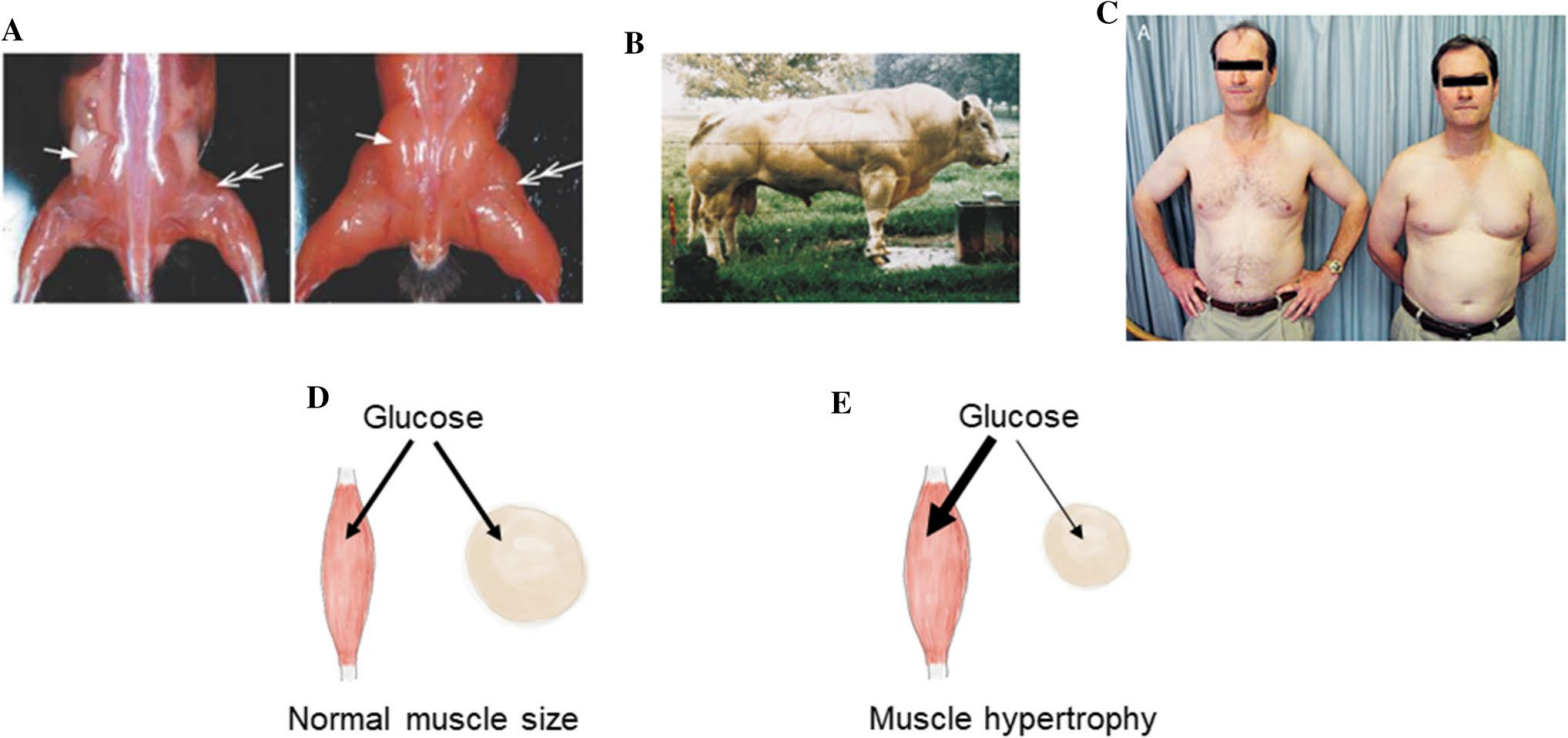
Indirect evidence for an association between muscle hypertrophy and low adiposity. **A** Loss of fat pads in mice with muscle hypertrophy where a muscle-specific HSA promoter drives the expression of constitutive Akt1-Egfp in muscle fibres. Even though the transgene is expressed in muscle, inguinal fat pads are lost [60]. **B** Belgian Blue bull with muscle hypertrophy due to a 11-nucleotide deletion of the *Mstn* gene that encodes the muscle mass inhibitor myostatin. Note the low amount of subcutaneous fat, suggesting again an association between muscle hypertrophy and low fat mass [64]. **C** Image of two monozygous twins where the right twin has hypogonadism because of a pituicytoma. The right twin had “proximal muscle wasting” but more body fat when compared with the unaffected twin on the left, again suggesting an inverse relation between muscle mass and fat mass [62]. **D**,** E** Schematic of a hypothesis explaining why muscle hypertrophy may result in leanness. When compared with muscles of normal size (**E**), more glucose and other small molecules are taken up by hypertrophying muscle and thus there is less glucose for triglyceride (fat) synthesis in white adipose tissue. This could possibly explain the effect of muscle hypertrophy on adipose tissue mass

Does a Warburg-like metabolic reprogramming of hypertrophying muscles have an anti-obesity effect? If hypertrophying muscles take up more glucose (Fig. 2A), then less glucose is available for lipid de novo biosynthesis by white adipose tissue. As a consequence, organisms with hypertrophying muscles should be leaner than organisms where muscle mass does not change or declines. This is often the case. For example, mice with muscle hypertrophy due to Akt1 gain-of-function [60] or myostatin loss-of-function [61] mutations are leaner than controls with normal muscle mass. Moreover, hypogonadal or castrated men are typically less muscular but have more adipose tissue and more frequently develop insulin resistance than non-hypogonadal men (Fig. 3 [62, 63]).

There is some evidence that this is also true for humans as a “myostatin boy” was not only more muscular but also appeared leaner than boys of a similar age [65]. Another study has shown that the induction of Akt1-induced hypertrophy in mice on a high-fat and high-sugar diet reduces fat and body mass [33]. Finally, treating obese older men with the hypertrophy-inducing anabolic steroids [41] decreases abdominal fat [66]. Indirect evidence comes from a systematic review that found that resistance training which induces muscle hypertrophy reduced fat percentage by 1.46% (confidence interval − 1.78 to − 1.14, *p* < 0.0001), body fat mass by 550 g (confidence interval − 750 to ≥ 340) as well as visceral fat [67]. More generally, individuals are commonly leaner when their muscles grow or when muscle mass is high (e.g. adolescents, young adults, bodybuilders) than when muscle mass is stable, low or declines (e.g. sarcopenic or testosterone-deficient individuals [68]). This is an observation with many caveats but worth exploring more systematically. Collectively, these observations suggest that muscle hypertrophy per se reduces white adipose tissue mass. It remains to be uncovered whether this is due to the metabolic reprogramming and elevated glucose and other small molecule uptake of hypertrophying muscles, which leaves less glucose for lipid synthesis by white adipose tissue. In addition, it has to be evaluated how the ageing process itself influences these reprogramming capacities.

## Summary and Conclusions

In this review, we discussed how proliferating, healthy and cancer cells reprogramme their metabolism to channel energy metabolites into anabolic reactions and provide substrates for epigenetic reactions such as methylation or acetylation. We also provided evidence that a hypertrophying muscle takes up more glucose and reprogrammes its metabolism, too, and that part of that glucose is diverted into anabolic pathways. Finally, we discussed evidence that hypertrophying muscles take up more glucose and other small molecules and that this can have anti-diabetic and anti-obesity effects. Importantly, muscle hypertrophy can have insulin resistance-improving and anti-obesity effects even if it is not triggered by glycogen-decreasing resistance exercise.

Key questions for future research are:

1. How does glucose uptake, flux and incorporation into biomass differ in-between a hypertrophying and non-hypertrophying skeletal muscle fibre?
2. Are anabolic treatments (resistance training, drugs) effective treatments for obesity and insulin resistance?
3. Is there an interconnected ageing triad of muscle atrophy (sarcopenia), hyperglycaemia and weight gain/obesity?
4. Do distinct muscle hypertrophy models differentially reprogramme energy metabolism and anabolism or is there a common metabolic muscle hypertrophy programme?
5. What signal transduction events are required for reprogramming metabolic genes in a hypertrophying skeletal muscle?
6. What energy metabolism enzymes or transporters limit muscle hypertrophy?
7. How much do circulating molecules such as glucose, glutamate and lactate contribute quantitatively to biomass in a hypertrophying muscle [10]?
